# Comparison of Dietary Kudzu Leaf Meal (*Pueraria montana* Var. *lobata*) and Alfalfa Meal Supplementation Effect on Broiler (*Gallus gallus domesticus*) Performance, Carcass Characteristics, and Organ Parameters

**DOI:** 10.3390/ani10010147

**Published:** 2020-01-16

**Authors:** Joseph P. Gulizia, Kevin M. Downs

**Affiliations:** School of Agriculture, Middle Tennessee State University, Murfreesboro, TN 37132, USA; kevin.downs@mtsu.edu

**Keywords:** kudzu, alfalfa meal, leaf meal, broilers, protein supplementation

## Abstract

**Simple Summary:**

The increase in demand for poultry products has directly influenced an increase in poultry production. This increase has placed a strain on the accessibility of high protein feed resources (e.g., soybean meal). High demands for these protein resources from large poultry production industries have left small-scale poultry producers with minimal access to these feed ingredients. In response to this demand, many small-scale poultry producers are utilizing plant leaf based ingredients (i.e., leaf meals) as a protein substitute. Leaf meals offer poultry producers a feed resource that is inexpensive and high in protein. Kudzu is a rapid growing invasive plant species that can engulf and destroy many environments. Kudzu contains a high concentration of nutrients, making it a potentially viable protein substitute in poultry production. In the present study, meat chickens (broilers) were fed a diet containing kudzu leaf meal to assess effects on broiler performance, carcass characteristics, and organ parameters during a 3 week growing period. The results indicated that broilers fed kudzu performed at a relatively high level. Utilizing kudzu leaf meal as a protein substitute could benefit poultry producers in tropical countries/regions where kudzu is highly accessible and other protein sources are not.

**Abstract:**

This research study was conducted to determine the effects of dietary supplementation of kudzu leaf meal (KLM) and alfalfa meal (AM) on broiler performance, carcass characteristics, and organ parameters. Kudzu leaf meal and AM were added at rates of 6% and 7.3%, respectively, to a complete broiler starter diet. Three treatments (control (complete broiler starter diet), KLM supplementation; and AM supplementation) with four replicates were fed to 217 male broilers over a 21 d battery cage grow out. Data were analyzed as a completely randomized design with battery cage representing the experimental unit. Birds on KLM and AM had a lower average body weight, lower cumulative feed consumption, and a higher adjusted feed conversion than control (*p* < 0.05). Additionally, there were observed treatment effects on whole breast weight (*p* = 0.0010), with control being higher than both treated diets. Minimal treatment effects were observed for organ parameters. Furthermore, there were no observed treatment differences for mortality (*p* > 0.05). Although broilers on KLM did not perform as well as those in the control group, these results are indicative that kudzu is safe to use in poultry production and has a high potential as a protein supplement in tropical regions with a low availability of commercial protein feedstuffs.

## 1. Introduction

There has been an increase in the demand for poultry products, thus increased poultry production, which has resulted in increasing demand for current proteinaceous feed resources such as soybean meal [1]. This has led to a decrease in the availability and increase in price of soybean meal, the most common protein source in poultry feeds [1,2]. In response, farmers worldwide are assessing economically viable alternative feedstuffs for poultry diets [1].

Alternative feed sources (e.g., leaf meals) are more commonly sought out by smallholders and large-scale poultry farmers where there are feed deficits and high feed costs [3,4]. Feed deficits and high prices of feed have made it difficult to obtain protein and energy sources in many regions [4]. Therefore, research has been conducted utilizing leaf meals as a potential dietary supplement in broiler production. Gudiso et al. [5] observed that supplementing *Acacia angustissima* (a tropical legume that originated in Central America) as a protein source in broiler diets could be used as an alternative, locally available protein for poultry industries in the tropics. Additionally, Ncube et al. [4] found that *Acacia angustissima* could be included in a diet up to 15% without deleterious effects. Gadzirayi et al. [6] studied the potential use of mature *Moringa oleifera* (a tropical or subtropical plant native to Africa and southern Asia) leaf meal as an alternative to soybean meal in poultry production. They concluded that *Moringa oleifera* leaf meal could be considered as a soybean meal supplement up to a 25% inclusion rate in broiler diets. Lastly, alfalfa meal (*Medicago sativa*) has been used in previous research as a feed supplement in table egg layer and broiler diets, and was a historically common protein source in commercial and home-mixed poultry feeds [7,8]. As a commercially available leaf meal, alfalfa meal is high in protein (up to 19% on a dry matter basis) but also high in crude fiber [7,9]. Therefore, it was used in this study as a standard of comparison when analyzing effects of kudzu leaf meal supplementation.

Kudzu (*Pueraria montana* var. *lobata*), an invasive semi-woody, perennial vine weed species native to eastern Asia was introduced to the United States in the late 1800s as a method of soil erosion control, but by the 1970s the United States Department of Agriculture (USDA) had placed it on the common weed list [10]. *Pueraria phaseoloides* (tropical kudzu) is prevalent in many tropical countries/regions as shown in Table 1. Kudzu crowds out and engulfs native species, preventing them from receiving necessary light to survive. Kudzu can grow in a wide range of soil types, including sandy, acid, lime, lowlands with high water tables, in over heavy subsoil, and in areas where winter soil temperatures do not drop below −32 °C [10,11]. It spreads quickly and grows rapidly, making it a problem for many environments worldwide.

Historically, kudzu has been used as a supplement in livestock diets; however, there is limited data on kudzu’s uses in poultry diets. Polk and Gieger [8] demonstrated that an inclusion rate of 9% kudzu leaf meal could be supplemented in place of alfalfa meal in chick diets. Additionally, Nworgu and Egbunike [24] fed *Pueraria phaseoloides* leaf meal (PLM) to broilers. They concluded that PLM should not be used while rearing broilers as it decreased growth rate. In contrast, Etela et al. [25] supplemented fresh *Pueraria phaseoloides* in the diets of Lohmann Browns (a chicken predominantly used as a layer) and suggested that leaf meals could be incorporated into poultry diets. Therefore, kudzu’s high nutrient composition could be a potential substitution for common, more expensive feed components typically used in broiler diets [26]. Leaf meals used as an alternative protein source have the potential to increase body weight and reduce feed costs in animal production [4,27,28,29]. In poultry diets, previous research has shown that optimum leaf meal supplementation varies with plant species and typically ranges from 4% to 15% supplementation [4,28,30,31]. Kudzu’s rapid growth and widespread availability allows easy access to use as a dietary supplement for a variety of animals.

Kudzu’s high concentration of nutrients is comparable to alfalfa (*Medicago sativa*), which was a historically common perennial legume used in poultry feeds [7,9,32]. Additionally, kudzu’s nutrient composition is comparable to *Acacia angustissima* and *Moringa oleifera*, which are current leaf meals being used as alternative protein sources in poultry production [33,34]. Other leaf meals comparable to kudzu’s nutrient composition are *Arachis pintoi*, *Crotalaria ochroleuca*, *Psophocarpus tetragonolobus*, and *Stylosanthes guianensis* [35,36,37,38,39]. Kudzu’s ready availability and leaf yield could be beneficial to producers searching for alternative protein sources for poultry feeds. Therefore, due to limited data on kudzu in poultry, this study will assess the potential viability of kudzu as a dietary supplement for modern commercial broilers. Results from this study could benefit poultry producers in tropical countries/regions with significant kudzu availability and a scarcity of high quality feedstuffs.

## 2. Materials and Methods

Kudzu collection and preparation: Kudzu used in this research was collected from seven different counties in the middle Tennessee area (Bedford, Cannon, Coffee, Dekalb, Lawrence, Maury, and Rutherford). Sample collection occurred from early June to late October. The method of collection used a 30.5 cm^2^ polyvinyl chloride (PVC) square randomly tossed into clusters of kudzu. Content within the square was collected by clipping an estimated 15 cm depth of kudzu vine. After collection, leaves were removed and dried at 60 °C for 48 h. Only kudzu leaves were used when supplementing KLM into the commercial broiler starter diet. Once dried, kudzu leaves were ground and passed through a 2 mm sieve then compiled into a composite sample across all counties.

Diet preparation: In this study, experimental treatments included control (basal diet), kudzu leaf meal (KLM) supplementation, and alfalfa meal (AM) supplementation (Table 2). The control diet was a proprietary complete commercial broiler starter diet (corn-soybean meal based) obtained from a local broiler complex. The starter feed used in each treatment met or exceeded National Research Council (NRC) requirements [40]. Supplementation of KLM and AM into the complete broiler starter diet (basal diet) was at rates of 6% and 7.3%, respectively. Different inclusion rates were to ensure that each supplemented diet contained similar amounts of plant-based protein. Fishmeal (TerraMar; 58% CP) was added at a 1% inclusion rate to both KLM and AM. Fish meal was added at equal rates to treated diets to ameliorate any effects from fish meal, while maintaining an isonitrogenous state between all treatments. The basal broiler starter feed contained 26.9% CP, thus each experimental treatment with an added supplement (i.e., kudzu leaf meal and alfalfa meal) was adjusted to ensure all treatments were isocaloric and isonitrogenous (Table 2). Additionally, all treatments were uniformity tested using the Quantab^®^ Chloride Titrator method to determine mixing performance. Herman and Behnke [41] and Stark and Saensukjaroenphon [42] classify mixer tests as excellent (<10%), good (10–15%), fair (15–20%), and poor (>20%) based on percent coefficient of variation (% CV). Kudzu leaf meal (8.8% CV) and AM (6.1% CV) were tested as mixed excellent, with the control (13.3% CV) being mixed at a level of good.

Nutritional analysis: Samples of kudzu leaf meal and alfalfa meal (alfalfa meal purchased from a commercially available source) were obtained and submitted to Dairy One Laboratory (Ithaca, NY) for near infrared reflectance spectroscopy (NIR) chemical composition analysis (Table 3). Both experimental feed ingredients were analyzed for DM, CP, ADF, NDF, Lignin, ME, Ca, P, Mg, K, S, Cl, Lysine, and Methionine. Additionally, diet samples from all treatments (i.e., control, kudzu leaf meal supplementation, and alfalfa meal supplementation) were obtained and submitted to Dairy One for nutrient analysis prior to study initiation (Table 2). All treatments were analyzed for DM, CP, ADF, NDF, ME, Ca, P, Mg, K, Na, Fe, Zn, Cu, Mn, and Mo. Both supplemental leaf meals and dietary treatments were analyzed according to AOAC and Van Soest fiber analysis procedures [43,44].

Broiler performance: Two-hundred and seventeen male Cobb 700 broilers (obtained from a local complex) were grown for 21 d in a battery cage study that compared the effects of KLM and AM supplementation on performance, carcass characteristics, and organ parameters. Chicks were vaccinated against Marek’s Disease with a ½ dose of HVT/CVI rispens. No other vaccinations were given after chicks left the hatchery.

In this study, each cage was provided ad libitum feed for each treatment from d 0–7, and all cages were feed restricted, to control growth, for 6 h per day from d 7–14 and reduced to 4 h per day from d 15–21. Treatments were randomly assigned to each cage (four replicate cages/treatment). Control and AM contained 72 birds/treatment, with KLM containing 73 birds/treatment. Eleven cages (0.79 m^2^/cage) contained 18 broilers (436.64 cm^2^/bird) and one cage (0.79 m^2^/cage) contained 19 broilers (415.79 cm^2^/bird) (totaling 12 experimental units), with birds randomly assigned to each cage. All birds had adequate feeder and drinker space. Birds were brooded at approximately 35 °C and reduced approximately 3 °C every 7 d. Room temperature was kept at 27 °C. Continuous light (24 L:0 D) was provided and water was offered ad libitum.

During this 21 d grow out, birds were weighed twice per week (d 3, 7, 9, 12, 14, 18, and 21) to determine average body weight gain. Feed consumption was determined by calculating the difference between feed offered and feed that remained on designated days birds were weighed. Cumulative feed consumption was determined by calculating the difference of feed offered and feed that remained between d 0–7, 0–14, and 0–21. Feed conversion (FCR) was calculated by using feed consumption and body weight gain (feed consumed/body weight gain = FCR). Feed conversion was adjusted for mortality. On d 21, feed was withdrawn 2 h before all broilers were humanely euthanized to assess carcass characteristics. Whole carcasses were chilled at approximately 1 °C in an ice water bath for 10 h. Whole breast was removed by cutting through the ribs and at the junction of the coracoid, scapula, and clavicle. Paws were removed below the spur. Whole breast and paws were removed to assess weight and yield of these carcass characteristics. The liver and gall bladder were excised from the bile duct. After removal, the gall bladder was then separated from the liver. The gizzard was removed at the proventricular-ventricular junction and the gizzard-duodenal loop junction. Both testes were separated from the vas deferens and removed from the abdominal cavity. Ceca were excised at the ileo-colonic cecal junction. Lastly, the entire small intestines were removed at the ileo-colonic cecal junction and gizzard-duodenal loop junction. The pancreas was removed from the duodenal loop. All organ parameters were removed to assess absolute weight and percent of chilled carcass weight.

Statistical analysis: Data were analyzed as a completely randomized design with battery cage representing the experimental unit. Treatment main effect significance for average body weight, adjusted feed conversion (AFCR), and mortality were determined using the general linear models procedure of the SAS statistical package [45]. In addition, chilled carcass weight, whole breast weight and yield, paws weight and yield, organ (liver, gizzard, testes, ceca, and small intestines) absolute weight and percent of chilled carcass weight were analyzed for treatment effect significance using the general linear models procedure of the SAS statistical package [45]. Means were separated using the PDIFF comparison method of SAS (*p* < 0.05). All data were analyzed for normality using the Shapiro–Wilk test. Animal handling procedures were approved by the Middle Tennessee State University Institutional Animal Care and Use Committee (IACUC) and conform to accepted practices [46,47].

## 3. Results

**Figure 1:**
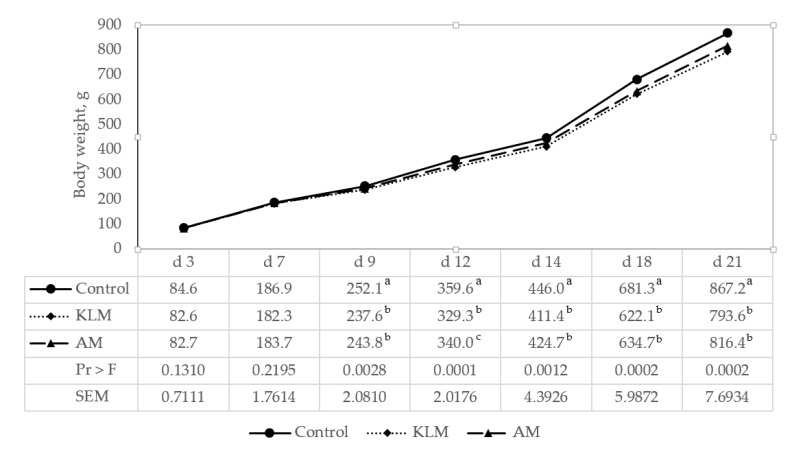
Influence of control (basal diet only), kudzu leaf meal supplementation (KLM (basal diet + 6% inclusion rate of kudzu leaf meal)), and alfalfa meal supplementation (AM (basal diet + 7.3% inclusion rate of alfalfa meal)) on body weight at d 3, 7, 9, 12, 14, 18, and 21 (least squares means). ^a–c^ Means in the same column with different superscript letters are significantly different (*p* < 0.05).

Average body weight: There were no significant treatment effects for average body weight (g/bird) on d 7 between control, KLM, and AM (*p* = 0.2195). However, significant treatment effects for average body weight were observed on d 14 (*p* = 0.0012) and 21 (*p* = 0.0002) (Table 4). Average body weight differences during the 21 d grow out are illustrated in Figure 1. On d 14 and 21, control birds had a higher average body weight than birds on KLM and AM (Table 4). Additionally, there were no significant treatment effects throughout the study for average body weight between KLM and AM.

Adjusted feed conversion: Between d 0 and 7, there were no significant treatment effects between control, KLM, and AM for AFCR (g:g) (*p* = 0.1385). However, there were significant treatment effects for AFCR for control, KLM, and AM between d 7–14, 0–14, 14–21, and 0–21 (*p* < 0.05) (Table 4). Overall, birds on the control diet had a lower AFCR than birds on KLM and AM throughout the study. Although the control birds had a lower AFCR, between d 14–21 and 0–21 there were no significant treatment effects between the control and AM diet (*p* > 0.05). Additionally, there were no significant differences for AFCR between KLM and AM throughout the study (Table 4).

Mortality: There were no significant treatment effects on mortality (%) between control, KLM, and AM for the 21 d grow out (*p* > 0.05) (Table 4).

Carcass characteristics: Significant treatment effects were observed for chilled carcass (chilled whole bird) weight (g/bird) and whole breast weight (g/bird), with birds in the control group having higher weights than birds fed KLM and AM (*p* = 0.0006 and *p* = 0.0010, respectively) (Table 5). Birds on KLM tended to have a higher paw yield (%) than birds in the control group (*p* = 0.0778). Overall there was not a significant effect among treatments for whole breast yield and paws yield (*p* > 0.05). In addition, there were no significant treatment effects for paws weight (g/bird) between control, KLM, and AM (*p* > 0.05). Birds on the control diet had a higher chilled carcass weight and whole breast weight than birds on KLM and AM (*p* < 0.05). There were no significant treatment effects for whole breast weight, whole breast yield, and paws yield between KLM and AM (*p* > 0.05) (Table 5).

Organ parameters: Organ parameters were assessed as absolute organ weight (g/bird) and organ as a percent of chilled carcass weight (%). Between the control, KLM, and AM diets, there were significant treatment effects on liver weight (LW), % liver (LW%), % gizzard (GW%), % ceca (CW%), and % small intestine (SIW%) (*p* < 0.05) (Table 6). Additionally, small intestines weight (SIW) tended toward significance, with birds on the control and AM diets having a higher weight than KLM (*p* = 0.0578). However, there were no significant treatment effects observed for gizzard weight (GW), testes weight (TW), % testes (TW%), and ceca weight (CW) between control, KLM, and AM (*p* > 0.05) (Table 6). Birds on the control diet had a significantly higher LW than birds on KLM and AM (*p* < 0.05). In addition, birds on the control diet had a significantly higher LW% than birds on AM (*p* < 0.05), but not birds on KLM (*p* > 0.05). Ceca (%) and SIW% were significantly greater for birds on KLM and AM than the control diet (*p* < 0.05); however, there were no significant treatment effects between KLM and AM on CW% and SIW% (*p* > 0.05). Birds on the KLM diet had a significantly higher GW% than the control diet and AM (*p* < 0.05) (Table 6).

## 4. Discussion

Average body weight: There were no significant treatment effects observed on d 7; however, birds fed KLM had a 7.8 and 8.5% lower average body weight than birds on the control diet on d 14 and 21, respectively. Nworgu and Egbunike [24] observed that weight gain decreased when supplementing increasing levels of tropical kudzu (*Pueraria phaseoloides*) in broiler diets. Overall, a number of studies have shown that a majority of leaf meals depress feed consumption, thus decreasing body weight gain. Gudiso et al. [5] concluded that a significant negative linear response on average daily feed intake and average body weight gain occurred when supplementing increasing levels of *Acacia angustissima* leaf meal as a protein substitute in broiler diets. High levels of leaf meals included in the diet could cause nutrient imbalances and inhibit metabolism, thus negatively affecting growth performance [5,48]. However, Etela et al. [25] observed that there were no treatment effects on total feed intake and total body weight gain between tropical kudzu and a control diet (commercial type broiler diet) throughout a 62 d grow out. Although traditional feedstuffs (i.e., corn and soybean meal) produce higher performing birds, using kudzu as a protein supplement appears to be a viable option due to birds on KLM still having adequate 21 d average body weight when compared to control and AM in the present study.

Implementing kudzu as a viable protein supplement in small, growing poultry farms, that have limited access to traditional feedstuffs, in tropical regions could benefit those smallholders in reducing expenses and increasing bird performance. Current research on leaf meals suggests that substituting costly modern protein feedstuffs with local forages could potentially provide a high quality protein supplement, while utilizing an inexpensive feedstuff. Gudiso et al. [5] reported that *Acacia angustissima* leaf meal has potential to provide an inexpensive viable protein supplement for poultry production in the tropics. In the current study, there was no observed differences between KLM and AM during the 21 d grow out. Similar results were observed by Polk and Gieger [8] with kudzu products (i.e., kudzu meal and kudzu leaf meal) being comparable to AM during a 9 week grow out.

Adjusted feed conversion: Feedstuffs that contain a high percentage of indigestible fiber cause a bulkiness effect in the digestive tract of broilers [4,5]. Bulky diets tend to decrease feed digestibility, increase impaction of the gastrointestinal tract, and dilute nutrients, which leads to a decrease in available nutrients and metabolizable energy [4,5]. High fiber diets that cause a bulkiness effect have the potential to negatively impact broiler performance (i.e., body weight gain and FCR) [4,5]. After d 7 in the present study, birds supplemented leaf meals experienced a fiber bulkiness effect, thus, contributing to their depressed adjusted feed conversion.

Significant treatment effects for AFCR were not observed until after d 7. Adjusted feed conversion for KLM and AM between d 0–14 and 7–14 were observed to be higher than birds fed control. Additionally, birds on the control diet had a lower AFCR than birds fed KLM, but not AM between d 0 and 21. Thus, birds that were fed the control diet were more efficient. Etela et al. [25] reported that birds supplemented with tropical kudzu experienced a higher FCR and lower protein efficiency than birds fed a commercial broiler diet. Research has observed that high fiber diets negatively affect broiler performance. These observations suggest that birds supplemented with leaf meals as a protein substitute will not utilize the nutrients as efficiently as other protein sources (e.g., soybean meal). Although birds fed KLM were significantly less efficient than birds on the control diet, KLM birds still performed at a relatively high level and are comparable to industry broiler performance standards [49].

Mortality: Previous research conducted by Polk and Gieger [8] demonstrated that mortality in birds fed diets containing kudzu products (i.e., kudzu leaf meal and kudzu meal) compared favorably to birds fed alfalfa meal over a 9 week study. Similarly, the present study observed that kudzu appears safe as a viable protein source for poultry feed as indicated by a lack of differences in mortality rate (%) between treatments.

Carcass characteristics: Significant treatment effects observed for whole breast weight corresponds to average body weight. Thus, birds fed control had a higher average body weight and whole breast weight than birds on KLM and AM on d 21. However, there was no overall (across all treatments) effect on whole breast yield. In the present study, supplemental dietary leaf meals did not affect whole breast yield, indicating that birds fed either KLM or AM could produce whole breast yields comparable to birds fed a commercial starter feed.

Overall, birds fed KLM and AM had a 0.17% and a 0.09%, respectively, higher paw yield than birds in the control group. Paw yield tended to be higher for birds fed supplemental dietary leaf meals. However, this observed effect is not correlated to paw size. Overall, paw yield effects were principally due to control birds having higher chilled carcass weights than KLM and AM birds. This is likely due to the rate of paw growth and muscle deposition being different. Although there were no observed supplemental dietary leaf meal treatment effects, further research may be warranted to assess the potential dietary leaf meal effects on paw growth.

Organ parameters: Organ parameters were assessed as absolute organ weight (g/bird) and organ as a percent of chilled carcass weight (%). Overall, birds on the control diet had a higher LW and LW% than birds fed KLM and AM. During a chicken’s first 8 weeks of life, Daghir and Pellett [50] have corresponded that organ weight and body weight do not change at the same rate. Thus, birds in this study in the control group do not have a higher LW and LW% due to higher average body weight. Akiba and Matsumoto [51] and Zaefarian et. al. [52] conducted extensive reviews of the liver and determined that liver weight was reduced from an increased inclusion rate of cellulose in the diet. This effect is potentially caused from an increased fiber content reducing liver lipid accumulation and plasma lipid content; however, this effect tends to be convoluted with determining if increased fiber content or decreased energy intake reduced liver lipid accumulation [52]. Additionally, previous research has linked the reduction of liver weight to an increased secretion of bile, thus the increased metabolic activity suppressed tissue deposition and growth of the liver [52,53,54,55]. This present study followed previous results indicating higher dietary fiber content affects the liver of broilers.

Birds fed KLM had a 0.25% higher GW% than birds on the control diet during the 21 d grow out. Etela et al. [25] observed similar outcomes when supplementing green foliage supplements (i.e., fresh centrosema (*Centrosema molle*), tropical kudzu (*Pueraria phaseoloides*), or waterleaf leaves (*Talinium triangulare*)) in broiler diets. The authors concluded that gizzard weight was higher when the diet consisted of supplemented green feeds. Extensive reviews on the function of the gizzard have determined an increase in gizzard size when the diet consists of structural plant components (i.e., coarse/fine fibers or cereals) [56,57]. Svihus [57] found that fibrous material causes a pH change in the gizzard environment which results in an increase in gizzard volume and ability to retain digesta for a longer period of time. In the present study, the increase in GW% of both leaf meal treatments is consistent with previous research showing an increase in GW and GW% occurs when there is an increased dietary fiber content.

Manipulations made to poultry diets have a substantial impact on the digestive tract [57]. The composition of the diet greatly affects the size of the ceca [57]. Ceca (%) was observed to be higher in birds fed KLM and AM than birds in the control group. As the percent of fermentable materials in the diet increases, the ceca will react by increasing in size [57]. Previous research observed the ceca increasing approximately 30% in length when chickens ate a fiber-rich diet during the winter [57,58]. Results are consistent with previous research showing that significant fibrous material in poultry diets will increase the ceca weight as a percent of carcass weight.

Significant treatment effects on SIW and SIW% were observed during the 21 d grow out. In this study, the observed small intestine effects could be directly related to KLM and AM containing more fibrous material and exhibiting more bulky characteristics than control. However, Svihus [57] determined that, due to the difficulty in assessing small intestine functionality, interpretation of effects on the small intestine is often complex and more accurately performed utilizing histological procedures. Therefore, further research is needed to accurately assess potential leaf meal effects on SIW and SIW%.

## 5. Conclusions

Kudzu leaf meal as a protein substitute for broilers was evaluated for its effects on broiler performance, carcass characteristics, and organ parameters. Reduction in body weight, feed consumption, and AFCR were observed when broilers were fed kudzu leaf meal at a 6% inclusion rate. Kudzu appears to be a safe alternative protein source for broilers as concluded from the mortality results. Although, kudzu leaf meal in this study had a high nutrient composition, the anti-quality/nutritional factors inherent to kudzu that could potentially affect animal performance are largely unknown. Further research is needed to assess those inherent effects. Additionally, more research on optimum inclusion rates of kudzu leaf meal are needed to assess potential bird production increases. Birds that consumed kudzu leaf meal had reduced performance when compared to a modern feeding program, however they still performed at a high level. Based on the results of this study, kudzu leaf meal could be considered as a potential protein supplement for poultry diets, particularly in world regions where kudzu is highly accessible while other protein feedstuffs are not.

## Figures and Tables

**Table 1:** Distribution of *Pueraria phaseoloides* (tropical kudzu) in tropical countries/regions [12,13,14,15,16,17,18,19,20,21,22,23].

Country/Region	Distribution	Origin
Belize	Present	Introduced
Bolivia	Present	Introduced
Brazil	Present	Introduced
Colombia	Present	Introduced
Costa Rica	Present	Introduced
Cuba	Present	Introduced
Dominican Republic	Present	Introduced
Ecuador	Present	Introduced
French Guiana	Present	Introduced
Galapagos Islands	Present	Introduced
Guyana	Present	Introduced
Guadeloupe	Widespread	Introduced
Haiti	Present	Introduced
Jamaica	Present	Introduced
Martinique	Widespread	Introduced
Panama	Present	Introduced
Peru	Present	Introduced
Puerto Rico	Present	Introduced
Saint Lucia	Present	Introduced
Suriname	Present	Introduced
Trinidad and Tobago	Present	Introduced
United States Virgin Islands	Present	Introduced

**Table 2:** Nutrient composition of control (basal diet only), kudzu leaf meal supplementation (KLM (basal diet + 6% inclusion rate of kudzu leaf meal)), and alfalfa meal supplementation (AM (basal diet + 7.3% inclusion rate of alfalfa meal)) fed to experimental broilers during a 21 d battery cage grow out (DM basis).

Analysis	Control	KLM	AM
Dry matter (DM), %	88.5	88.4	88.7
Crude protein (CP), %	26.9	27.9	27.1
Adjusted crude protein, %	26.9	27.9	27.1
Acid detergent fiber (ADF), %	5.3	5.9	5.4
Neutral detergent fiber (NDF), %	8.6	10.8	11.4
Metabolizable energy (ME), Kcal/kg	3540.0	3520.0	3500.0
Ca, %	0.89	1.10	1.00
P, %	0.70	0.73	0.73
Mg, %	0.17	0.19	0.19
K, %	1.04	1.08	1.15
Na, %	0.145	0.145	0.139
Fe, ppm	138	139	183
Zn, ppm	148	130	137
Cu, ppm	148	137	128
Mn, ppm	203	202	187
Mo, ppm	1.9	2.3	2.4

**Table 3:** Near infrared reflectance spectroscopy (NIR) chemical composition of kudzu leaf meal and alfalfa meal added to a complete broiler starter diet (basal diet) (DM basis).

Analysis	Kudzu Leaf Meal	Alfalfa Meal
DM, %	89.7	91.3
CP, %	25.9	20.9
Available protein, %	23.5	18.6
ADF, %	25.5	35.3
NDF, %	41.9	46.1
Lignin, %	4.9	7.3
ME, Kcal/kg	2520.0	2110.0
Ca, %	3.44	1.49
P, %	0.24	0.26
Mg, %	0.32	0.26
K, %	0.93	1.67
S, %	0.28	0.30
Cl, %	0.61	0.17
Lysine, %	1.01	1.06
Methionine, %	0.35	0.33

**Table 4:** Influence of control (basal diet only), kudzu leaf meal supplementation (KLM (basal diet + 6% inclusion rate of kudzu leaf meal)), and alfalfa meal supplementation (AM (basal diet + 7.3% inclusion rate of alfalfa meal)) on broiler live performance (least squares means).

Item	Control	KLM	AM	Pr > F	SEM ^A^
Average b.w., g/bird					
Day 7	186.9	182.3	183.7	0.2195	1.7614
Day 14	446.0 ^a^	411.4 ^b^	424.7 ^b^	0.0012	4.3926
Day 21	867.2 ^a^	793.7 ^b^	816.4 ^b^	0.0002	7.6934
Cumulative feed consumption, g/bird					
Day 0 to 7	159.94	154.58	151.72	0.1429	2.6739
Day 0 to 14	511.40 ^a^	485.78 ^b^	496.11 ^b^	0.0068	4.2586
Day 0 to 21	1130.57 ^a^	1073.22 ^b^	1091.93 ^b^	0.0024	8.1829
Adjusted feed conversion ^B^, g:g					
Day 0 to 7	1.13	1.13	1.11	0.1385	0.0074
Day 7 to 14	1.37 ^a^	1.48 ^b^	1.45 ^b^	0.0053	0.0184
Day 0 to 14	1.28 ^a^	1.35 ^b^	1.34 ^b^	0.0453	0.0159
Day 14 to 21	1.49 ^a^	1.55 ^b^	1.54 ^a,b^	0.0984	0.0190
Day 0 to 21	1.39 ^a^	1.45 ^b^	1.43 ^a,b^	0.0275	0.0137
Mortality, %					
Day 0 to 7	0.00	1.32	4.17	0.2648	1.7131
Day 7 to 14	1.39	2.70	1.39	0.7659	1.4491
Day 0 to 14	1.39	4.02	5.56	0.5898	2.8154
Day 14 to 21	1.39	0.00	0.00	0.4053	0.8019
Day 0 to 21	2.78	4.17	5.56	0.8290	3.1739

^A^ Standard error of the mean; ^B^ Adjusted for mortality; ^a,b^ Means in the same row with different superscript letters are significantly different (*p* < 0.05).

**Table 5:** Influence of control (basal diet only), kudzu leaf meal supplementation (KLM (basal diet + 6% inclusion rate of kudzu leaf meal)), and alfalfa meal supplementation (AM (basal diet + 7.3% inclusion rate of alfalfa meal)) on carcass parameters (least squares means).

Item	Control	KLM	AM	P_r_ > F	SEM ^A^
Chilled carcass ^B^ wt., g/bird ^C^	894.10 ^a^	822.96 ^b^	850.90 ^c^	0.0006	8.0539
Whole breast ^D^ wt., g/bird ^C^	220.26 ^a^	195.69 ^b^	203.28 ^b^	0.0010	3.1200
Whole breast yield, % ^E^	24.64 ^a^	23.78 ^b^	24.05 ^a,b^	0.1183	0.2658
Paws wt., g/bird ^C^	25.61	24.30	24.94	0.1927	0.4626
Paws yield, % ^E^	2.86 ^a^	3.03 ^b^	2.95 ^a,b^	0.0778	0.0420

^A^ Standard error of the mean; ^B^ Chilled whole bird; ^C^ Average weight per chilled carcass; ^D^ Whole breast = skinless, bone-in *Pectoralis major* and *minor*; ^E^ Percent of chilled whole bird; ^a–c^ Means in the same row with different superscript letters are significantly different (*p* < 0.05).

**Table 6:** Influence of control (basal diet only), kudzu leaf meal supplementation (KLM (basal diet + 6% inclusion rate of kudzu leaf meal)), and alfalfa meal supplementation (AM (basal diet + 7.3% inclusion rate of alfalfa meal)) on organ parameters (least squares means).

Item	Control	KLM	AM	P_r_ > F	SEM ^A^
Liver wt., g/bird ^B^	25.35 ^a^	22.27 ^b^	22.55 ^b^	0.0017	0.4557
Liver wt., % ^C^	2.84 ^a^	2.71 ^b^	2.61 ^b^	0.0053	0.0342
Gizzard wt., g/bird ^B^	17.45	18.10	17.27	0.2208	0.3271
Gizzard wt., % ^C^	1.95 ^a^	2.20 ^b^	2.04 ^a^	0.0067	0.0413
Testes wt., g/bird ^B^	0.1406	0.1300	0.1363	0.4446	0.0057
Testes wt., % ^C^	0.0157	0.0158	0.0159	0.9831	0.0006
Ceca wt., g/bird ^B^	7.69	8.06	8.00	0.4566	0.2128
Ceca wt., % ^C^	0.8600 ^a^	0.9787 ^b^	0.9457 ^b^	0.0054	0.0195
Small intestines wt., g/bird ^B^	29.86 ^a^	28.12 ^b^	29.79 ^a^	0.0578	0.4936
Small intestines wt., % ^C^	3.34 ^a^	3.42 ^a,b^	3.52 ^b^	0.0180	0.0366

^A^ Standard error of the mean; ^B^ Average weight per chilled carcass (chilled whole bird); ^C^ Percent of chilled whole bird; ^a,b^ Means in the same row with different superscript letters are significantly different (*p* < 0.05).
